# The potential antimalarial efficacy of hemocompatible silver nanoparticles from *Artemisia* species against *P*. *falciparum* parasite

**DOI:** 10.1371/journal.pone.0238532

**Published:** 2020-09-01

**Authors:** Elisabetta Avitabile, Nina Senes, Cristina D’Avino, Ioannis Tsamesidis, Alessandra Pinna, Serenella Medici, Antonella Pantaleo

**Affiliations:** 1 Department of Biomedical Sciences, University of Sassari, Sassari, Italy; 2 Department of Chemistry and Pharmacy, University of Sassari, Sassari, Italy; 3 UMR 152 Pharma-Dev, Université de Toulouse, Toulouse, France; 4 Department of Materials, Imperial College London, London, United Kingdom; Universiti Malaysia Perlis, MALAYSIA

## Abstract

Malaria represents one of the most common infectious diseases which becoming an impellent public health problem worldwide. Antimalarial classical medications include quinine-based drugs, like chloroquine, and artesunate, a derivative of artemisinin, a molecule found in the plant *Artemisia annua*. Such therapeutics are very effective but show heavy side effects like drug resistance. In this study, “green” silver nanoparticles (AgNPs) have been prepared from two *Artemisia* species (*A*. *abrotanum* and *A*. *arborescens*), traditionally used in folk medicine as a remedy for different conditions, and their potential antimalarial efficacy have been assessed. AgNPs have been characterized by UV-Vis, dynamic light scattering and zeta potential, FTIR, XRD, TEM and EDX. The structural characterization has demonstrated the spheroidal shape of nanoparticles and dimensions under 50 nm, useful for biomedical studies. Zeta potential analysis have shown the stability and dispersion of green AgNPs in aqueous medium without aggregation. AgNPs hemocompatibility and antimalarial activity have been studied in *Plasmodium falciparum* cultures in *in vitro* experiments. The antiplasmodial effect has been assessed using increasing doses of AgNPs (0.6 to 7.5 μg/mL) on parasitized red blood cells (pRBCs). Obtained data showed that the hemocompatibility of AgNPs is related to their synthetic route and depends on the administered dose. *A*. *abrotanum*-AgNPs (1) have shown the lowest percentage of hemolytic activity on pRBCs, underlining their hemocompatibility. These results are in accordance with the lower levels of parasitemia observed after *A*. *abrotanum*-AgNPs (1) treatment respect to *A*. *arborescens*-AgNPs (2), and AgNPs (3) derived from a classical chemical synthesis. Moreover, after 24 and 48 hours of *A*. *abrotanum*-AgNPs (1) treatment, the parasite growth was locked in the ring stage, evidencing the effect of these nanoparticles to hinder the maturation of *P*. *falciparum*. The anti-malarial activity of *A*. *abrotanum*-AgNPs (1) on pRBCs was demonstrated to be higher than that of *A*. *arborescens*-AgNPs (2).

## Introduction

Malaria represents one of the most diffused human diseases caused by the mosquito bite, which is able to generate infection by introducing different species of *Plasmodium* into the host [1]. Among them, *P*. *falciparum* represents the most dangerous and lethal parasite infecting humans [2]. However, by considering the current drugs accessible for the prevention and treatment of this disease, it seems that, despite the effectiveness of available treatments, the main threat connected to malaria is the emergence of drug resistance [3, 4]. In this context, nanotechnology could represent a possible future solution against malaria drug resistance by introducing the control of drug release at the nanoscale or building active nanoparticles to be used against the parasite. In the last decades, the applications of nanotechnology have been mainly focused on the development of a large variety of nanoscale tools designed for their use in therapy [5, 6], leading to the improvement of drug delivery strategies in order to overcame the barriers found in several conventional therapeutics [7–9]. Among different nanomaterials, metal nanoparticles have been significantly studied thanks to their particular physical and chemical properties as alternative theranostic tools for treating a wide gamut of human diseases [10–12]. Malaria can be one of the targets of these new strategic weapons.

In this context, silver nanoparticles represent an ideal material for biomedical applications and have been largely investigated during the last years [13–15]. The use of silver remedies in medicine has been a common practice until the past century, when it ceased with the discovery of antibiotics in the 1940’s. The development of bacterial resistance to antibiotics, to which silver seems to be immune, renewed the interest toward this metal, and in the last few decades a large variety of silver compounds has been prepared, especially as coordination complexes, which also uncovered the great potential of silver as an anticancer, antifungal, antiparasitic and antimalarial agent [16]. Regrettably, such compounds have been successful in *in vitro* experiments only, with a scarce possibility of applications *in vivo* due to the low bioavailability of the active species (the Ag^+^ ion) inside the organism when they were transferred to animal models [16]. Still, the activity of silver-based drugs seems to be highly desirable, and the employment of AgNPs could bypass bioavailability problems, since they can act a reservoir of Ag^+^ ions inside the cell, released close to the molecular targets [17]. According to the literature, silver nanoparticles are toxic for prokaryotic organisms [18], but relatively safe for eukaryotic species, including humans. Their cytotoxicity is associated to several characteristics, including reactivity in solution, size distribution, shape, coating/capping, etc., which in turn depend on the synthetic method used for their preparation [19]. Indeed, conventional physical and chemical strategies for AgNPs synthesis are rather expensive, and reagents and solvents can be toxic [20, 21]. Thus, the preparation of eco-friendly metal nanoparticles using biological fluids represents a field of technology for nanomedicine applications which is growing with impressive speed [22]. Generally, extracts from plants, bacteria and algae, plant-based phytochemicals, and other biological sources are used as the reducing agents in the synthesis of metal nanoparticles with high stability and low toxicity [23–27].

The genus *Artemisia*, mainly diffused in the Northern hemisphere temperate regions, is one of the widest genera in the Asteraceae family, including more than 500 species, some of which have been traditionally used as folk remedies for the most disparate conditions, going from fever to common infections, intestinal parasites, hepatitis, and malaria. The therapeutic action of this genus of plants seems to be connected to the presence of a large variety of terpenes and thujones found in their extracts, which are currently being studied for the same applications [28]. *Artemisia annua* is actually the source of artemisinin, which is one of the reference compounds, together with its derivative artesunate, in the antimalarial treatment, but also *A*. *absinthium*, *A*. *afra*, *A*. *herba-alba*, *A*. *sieberi* have been and are still widely used as antimalarial remedies. *A*. *abrotanum*, a plant probably native to Southwestern Europe and diffused in the temperate regions of Europe, Western Asia (Turkey and Armenia) and North America, also known as “southernwood”, has been employed in traditional medicine for treating a variety of disorders, including upper airway diseases, but it also showed interesting antimalarial properties [29]. In a similar way, *A*. *arborescens*, a common species in ruderal environments on calcareous soils in the Mediterranean area, also known as “great mugwort” or “arborescent mugwort, is still used as an anti-inflammatory folk remedy [30]; moreover, it was traditionally employed on the island of Sardinia as an infusion against malarial fevers and other diseases before the advent of modern drugs. Furthermore, several studies have reported the potential of *A*. *arborescens* compounds as antibacterial, anti-inflammatory, antioxidant and anticancer agents [30–32]. Following these studies, an idea emerged as to investigate and compare the effects of AgNPs grown from *A*. *abrotanum* and *A*. *arborescens* against malaria.

As a matter of fact, many biologically active AgNPs have been prepared from other *Artemisia sp*. extracts. They all showed good antibacterial, anticancer and antifungal properties [33–37]. Anyway, to the best of our knowledge, *A*. *abrotanum* extracts have been employed in the synthesis of magnesium oxide (MgO) [38] or palladium (Pd) [39] nanoparticles for catalytic purposes only, while *A*. *arborescens* has never been used to prepare metal nanoparticles. Considering all the advantages of “green” AgNPs and the medicinal properties of *Artemisia* plants, especially against malaria, the synthesis of silver nanoparticles here proposed has been carried out using these two different *Artemisia* species, *A*. *abrotanum* and *A*. *arborescens*, and the AgNPs thus prepared have also been compared to AgNPs from a traditional chemical reduction for their activity against *P*. *falciparum* parasite.

## Material and methods

Unless otherwise stated, all materials were obtained from Sigma-Aldrich, St. Louis, MO, USA: silver nitrate, sodium citrate tribasic dehydrate, sodium citrate, ammonium hydroxide solution, ethanol. *A*. *abrotanum* plants were purchased from an accredited plant grower in Pulia.

*A*. *arborescens* leaves were collected in a growth spot in the countryside around Sassari (Sardinia, Italy). Both species were identified by a botanist of the Department of Chemistry and Pharmacy, University of Sassari.

### Cultivation of *Plasmodium falciparum*-infected RBCs

Freshly drawn blood (Rh+) from healthy adults of both sexes was used. Patients provided written, informed consent in ASL. 1-Sassari (Azienda Sanitaria Locale.1-Sassari) center before entering the study. This study was conducted in accordance with Good Clinical Practice guidelines and the Declaration of Helsinki. No ethical approval has been requested as Human blood samples were used only to sustain the parasites in vitro cultures. Blood anti-coagulated with heparin was stored in citrate-phosphate-dextrose with adenine (CPDA-1) prior to use. RBCs were separated from plasma and leukocytes by washing three times with RPMI 1640 medium. As previously reported, *Plasmodium falciparum* laboratory strain Palo Alto (PA), FCB1, It-G and ARS1 (all of them mycoplasma-free) were grown according to standard protocols [40, 41]. The Palo Alto (PA) strain represents a reference parasite strain to study various antimalarial drugs in *P*. *falciparum* [42, 43]. PA strain was isolated from a Ugandan patient and is considered as a reference strain due to its high genetic stability [41, 43]. Fresh red blood cells were selected and subsequently infected by the parasite of PA strain in order to create a continuous parasite culture of pRBCs to be used for treatments. *P*. *falciparum* PA strain (mycoplasma-free) was cultivated in RPMI 1640 medium containing HEPES, supplemented with 20 mM glucose, 2 mM glutamine, 0.025 mM adenine, and 32 mg/L gentamycin at 2% hematocrit. Parasite cultures were synchronized as described by Lambros and Vanderberg [42]. Throughout this procedure, *P*. *falciparum* cultures maintained synchronicity for 2–3 cycles.

### Synthesis of silver nanoparticles

Fresh *A*. *abrotanum* and *A*. *arborescens* leaves were collected in full thriving stage, in the months of February and March. Silver nanoparticles have been prepared using a modified version of the protocol reported in the literature by Khatoon *et al*. [44]. Fresh leaves were washed to remove dust and dirt, dried on paper, then weighed (10 g), manually minced and extracted (150 mL ethanol: water 1:1) for 30 minutes at the temperature of 50 °C, to avoid possible degradation of bioactive molecules. The extracts were then filtered and used to reduce silver nitrate to AgNPs. In details, 100 mL of the hydroalchoolic extracts were diluted to 500 mL with milliQ water and slowly added to 500 mL water solution of AgNO_3_ (340 mg) to reach a final molarity of 2 mM, under magnetic stirring at room temperature. Reaction was complete within a day. Silver nanoparticles have also been synthesized through a classical chemical approach (0.1 M AgNO_3_, 0.3 mM ascorbic acid as the reductant and 0.3 mM sodium citrate as the stabilizer, at 30 °C; the pH was adjusted to 13 with NH_4_OH to ensure a relatively small size of the synthesized nanoparticles) [45] and used for comparison. All AgNPs were recovered by centrifugation at 5000 rpm for 15 minutes. The yield of the reaction depended on the number of centrifuge cycles performed, and after 6 cycles about 60% of the initial silver was recovered, while the rest remained in suspension.

### Characterization of nanoparticles

The plasmonic peak of AgNPs was revealed by UV-Vis spectrum of the nanoparticles dispersed in aqueous buffer samples (1 mg/mL). Spectra were recorded by a Nicolet Evolution 300 UV-Vis spectrophotometer. The determination of the average size distribution of the nanoparticles by dynamic light scattering was performed using the Zeta sizer Nano-S90 (Malvern Panalytical) by dispersing the dried powder (1 mg) in water (1 mL). The zeta potential (ζ) of AgNPs was measured using a Zetasizer Nano ZSP (Malvern Instruments) in backscatter configuration (θ = 173°; laser wavelength of λ = 633 nm). The scattering cell temperature was fixed at 298 K, and the data were analysed through the Zetasizer software 7.03 version. Samples were prepared by suspending AgNPs (1 mg/mL) in milliQ water, left under rotation for one hour and sonicated for 20 min before analysis. Samples for Fourier Transform Infrared Spectroscopy were prepared as KBr pellets using nanoparticles or dried plant extracts (1% m/m). FTIR measurement were performed by recording the signals in the 400–4000 cm^-1^ range with a resolution of 4 cm^-1^ on a Vertex 70 Bruker spectrophotometer and analysed with OPUS 7 software. The X-ray diffraction analysis (XRD) was performed on a diffractometer (SmarLab model from Rigaku) aligned in the symmetrical Bragg-Brentano configuration with a Cu rotating anode source (lambda = 1.5418 A) and a graphite monochromator in the diffracted beam. Patterns were collected in the angular range from 25° to 120° in 2-theta, with a step-size of 0.05°, counting for 4 sec at each step. The Transmission Electron Microscopy analysis (TEM) were performed on FEI TECNAI G2 F20 TWIN instrument with an accelerating voltage of 200 kV. The Energy Dispersive X-ray Spectroscopy (EDX) was employed to collect the spectrum of elemental composition. Samples for TEM and EDX analyses were prepared by dispersing a small amount of nanoparticles in ethanol, sonicating them for 20 or 30 min, followed by the deposition of one or two drops of the suspension on a holey carbon/copper supported grid.

### Hemolysis assay on pRBCs cultures

The pRBCs cultures previously obtained were provided in order to perform the hemolysis assay. Fresh RBCs were prepared to maintain the pRBCs cultures at 1% haematocrit. Fresh human heparinized whole blood was obtained from healthy volunteer donors. RBCs were purified from blood by centrifugation at 200 g for 5 min to remove plasma and leukocytes. RBCs were then washed three times in sterile complete growth medium as previously described [40–42]. Parasitized RBCs cultures were maintained at 2–5% parasitemia (1% haematocrit) at 37 °C in a 95/5% (vol/vol) air/CO_2_-atmosphere. All assays were performed at this parasitemia and hematocrit. Hemolysis buffer (5 mmol/L sodium phosphate, 1 mmol/L EDTA, pH 8.0) was used as the positive control (Ctrl+), while PBS 1X and 5 mM glucose were used as the negative control (Ctrl-). To determine the hemolytic activity on pRBCs, nanoparticles suspension (stock = 1 mg/mL) prepared with sterile isotonic PBS 1X and 5 mM glucose was added to diluted pRBC culture (0.1 mL, ~ 2 × 10^8^ cells/mL) at different concentrations (0.6, 1.25, 2.5, 5, 7.5 μg/mL) for 24 and 48 hours of incubation at 37 and 41 °C (Thermomixer). Then, samples were centrifuged at 200 g for 1 minute and a microplate reader (Thermo Scientific) was used to measure the absorbance of hemoglobin release in the supernatant. The absorbance value of hemoglobin at 600 nm was measured with the reference wavelength of 405 nm. The percent of hemolysis was calculated as follows: Hemolysis % = [(sample absorbance _ negative control)/ (positive control _ negative control)] _ 100%.

### Silver nanoparticles susceptibility assays of cultured parasites

1 mg/mL of silver nanoparticles was serially diluted prior to addition to malaria cultures in PBS 1X and 5 mM glucose. Untreated cultures were run in parallel with the same final concentration. Cultures at the ring stage and fresh isolates of *P*. *falciparum* were treated for 24 and 48 hours in the presence of the indicated concentrations of all the tested silver nanoparticles: *A*. *abrotanum*-AgNPs (1), *A*. *arborescens*-AgNPs (2), AgNPs (3). All compounds were freshly prepared before any experiment and used immediately.

### Assessment of parasitemia by light microscopy

The morphology and the total parasitemia assays were performed using a standard method [40–42]. Parasite viability and parasitemia of PA strain were determined using Diff-Quick stained thin blood smears and light microscopy (Carl Zeiss Standard Microscope Lamphouse 467230). Parasitemia was defined as the number of parasites/number of RBCs counted, for a total of 5000 RBCs. Two thin smears per condition were counted 3 separate times by each of three operators. Cultures were synchronized weekly using Percoll separation or 5% sorbitol solution treatment [42] in order to obtain the first parasite stage (rings) to start the experiments at 0 h. The experiments were carried out at least in triplicate.

### IC_50_ measurement

To calculate the half maximal inhibitory concentrations of the different silver nanoparticles, ICEstimator software version 1.2 was used. The program estimates IC_50_ values using a nonlinear regression function of the R software.

### Statistical analysis

Data analyses were performed using Prism GraphPad software. Statistics for experiments were performed using a t-test. In all cases * was used for p < 0.05, ** for p < 0.01, and *** for p < 0.001. Values were expressed as the mean ± SD. All experiments were performed at least in triplicate.

## Results

### Characterization of “green” nanoparticles

**Fig 1:**
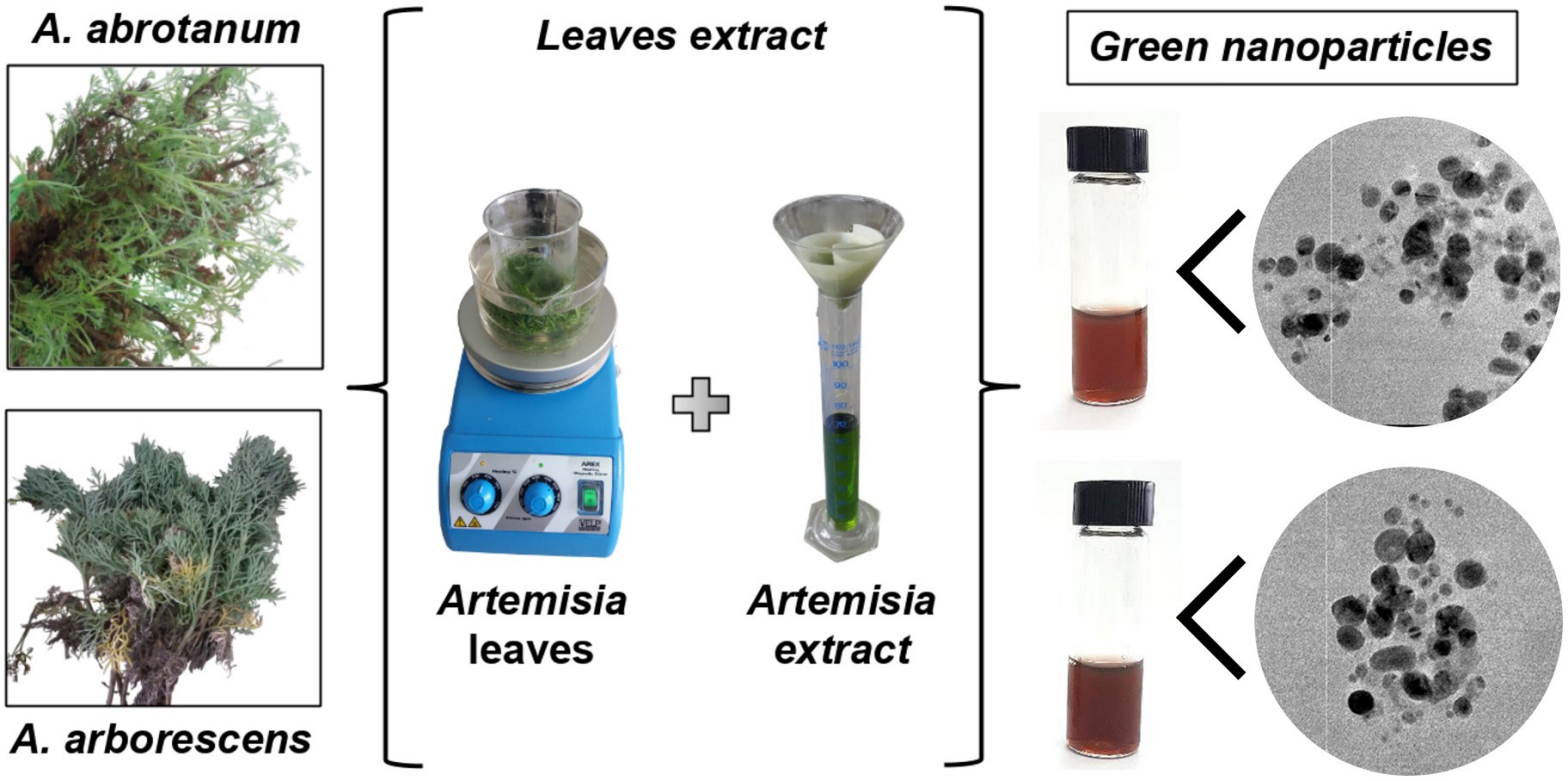
Green nanoparticles. Schematic representation of “green” AgNPs synthesis using *Artemisia* leaves extracts.

“Green” AgNPs have been prepared from leaf extracts using a modified method [44] for analogous AgNPs from *A*. *annua* (different extracts-to-silver ratio) as summarised in (Fig 1), which shows the synthetic process using the two different species of *Artemisia* plants. This change was introduced in the attempt of preparing particles with a small size (under 50 nm) useful for biological applications [46]. However, we need to consider t that AgNPs can cause hemolysis, which was found to be size- and dose-dependent, being higher for very small nanoparticles [47]. Cell uptake is also correlated to the nanoparticle size, being optimal for middle-sized AgNPs, around 50 nm [47].

**Fig 2:**
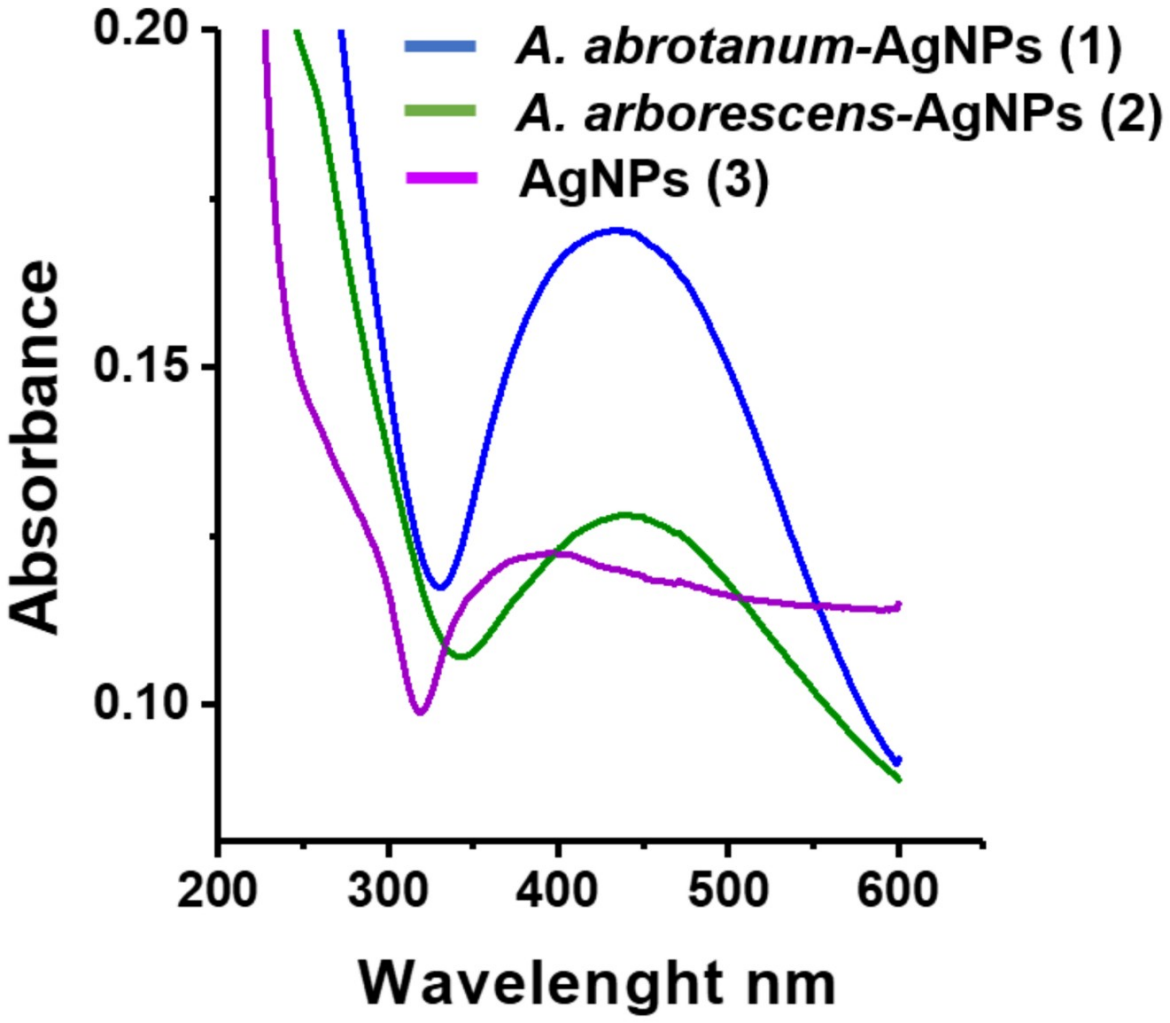
Characterization of nanoparticles surface plasmon resonance. Uv-vis spectra of *A*. *abrotanum*-AgNPs (1) (blue line), *A*. *arborescens*-AgNPs (2) (green line) and AgNPs (3) (violet line).

**Fig 3:**
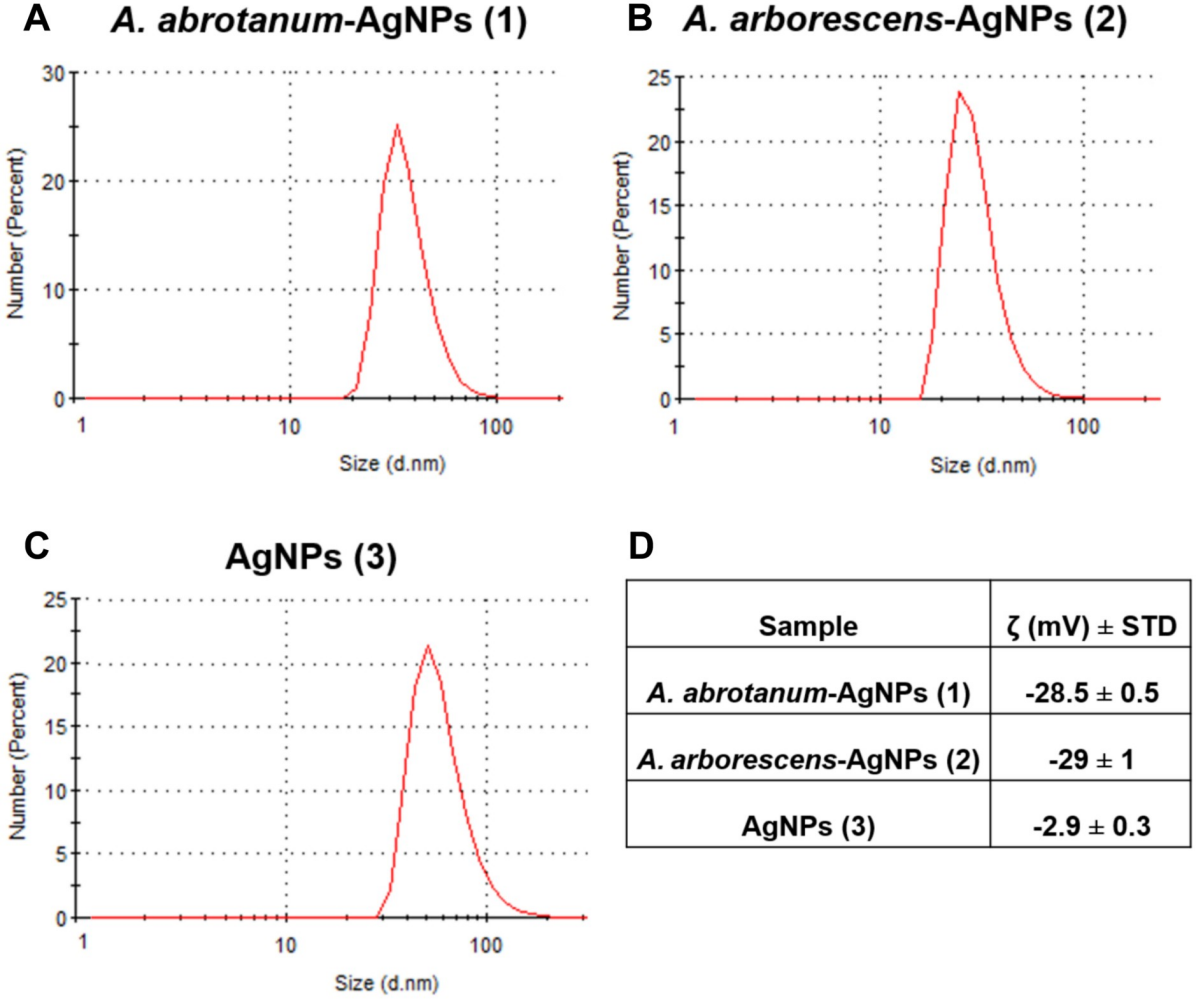
Nanoparticles characterization. Evaluation of nanoparticle size distribution using dynamic light scattering analysis; A) *A*. *abrotanum*-AgNPs (1) 37 nm, B) *A*. *arborescens*-AgNPs (2) 30 nm and C) AgNPs (3) 60 nm. D) Zeta Potential value for stability and dispersion in aqueous medium of nanoparticles. All “green” nanoparticles were well dispersed in aqueous medium showing no aggregation in solution.

The characterization of AgNPs synthetized using *Artemisia* leaf extract was carried out by a series of spectroscopic analyses. The UV-Vis spectra show the AgNPs surface plasmon resonance effect which reflects the method of synthesis, in turn affecting nanoparticle shape and size. Data results indicate that the peak observed in the range 400–450 nm was increased in *A*. *abrotanum*-AgNPs (1) and *A*. *arborescens*-AgNPs (2) respect to “classical” AgNPs (3), suggesting a spheroidal shape for these nanoparticles with a size below 50 nm [48] (Fig 2). Contrarily, “classical” AgNPs (3) displayed a lower absorbance intensity, probably due to their aggregation in solution (Fig 2). Dynamic light scattering analyses were used to evaluate the size distribution of AgNPs in aqueous dispersion. The average hydrodynamic size of “green” nanoparticles was around 37 nm for *A*. *abrotanum*-AgNPs (1), and 30 nm for *A*. *arborescens*-AgNPs (2), respectively (Fig 3A and 3B). All “green” nanoparticles were well dispersed in aqueous medium showing no aggregation in solution. On the other hand, AgNPs (3) from the classical chemical reduction appeared to be rather aggregated. However, dynamic light scattering analyses revealed their average size to be around 60 nm (Fig 3C). In order to describe the nanoparticles stability in acqueous medium, zeta potential analysis was performed. *A*. *abrotanum*-AgNPs (1) and *A*. *arborescens*-AgNPs (2) have shown a good zeta potential value (Fig 3D) underlining their good dispersion in aqueous medium without aggregation (S2 and S3 Figs). Contrariwise, “classical” AgNPs (3) displayed a low zeta potential value (-2.9 ± 0.3) (Fig 3D and S4 Fig) evidencing their propensity to aggregation in accordance with the other characterization results reported.

### Evaluation of “green” nanoparticles surface

The evaluation of the structural features of the nanoparticle surface plays a significant role in the understanding of the possible effects of AgNPs on the cell membrane for biological applications.

**Fig 4:**
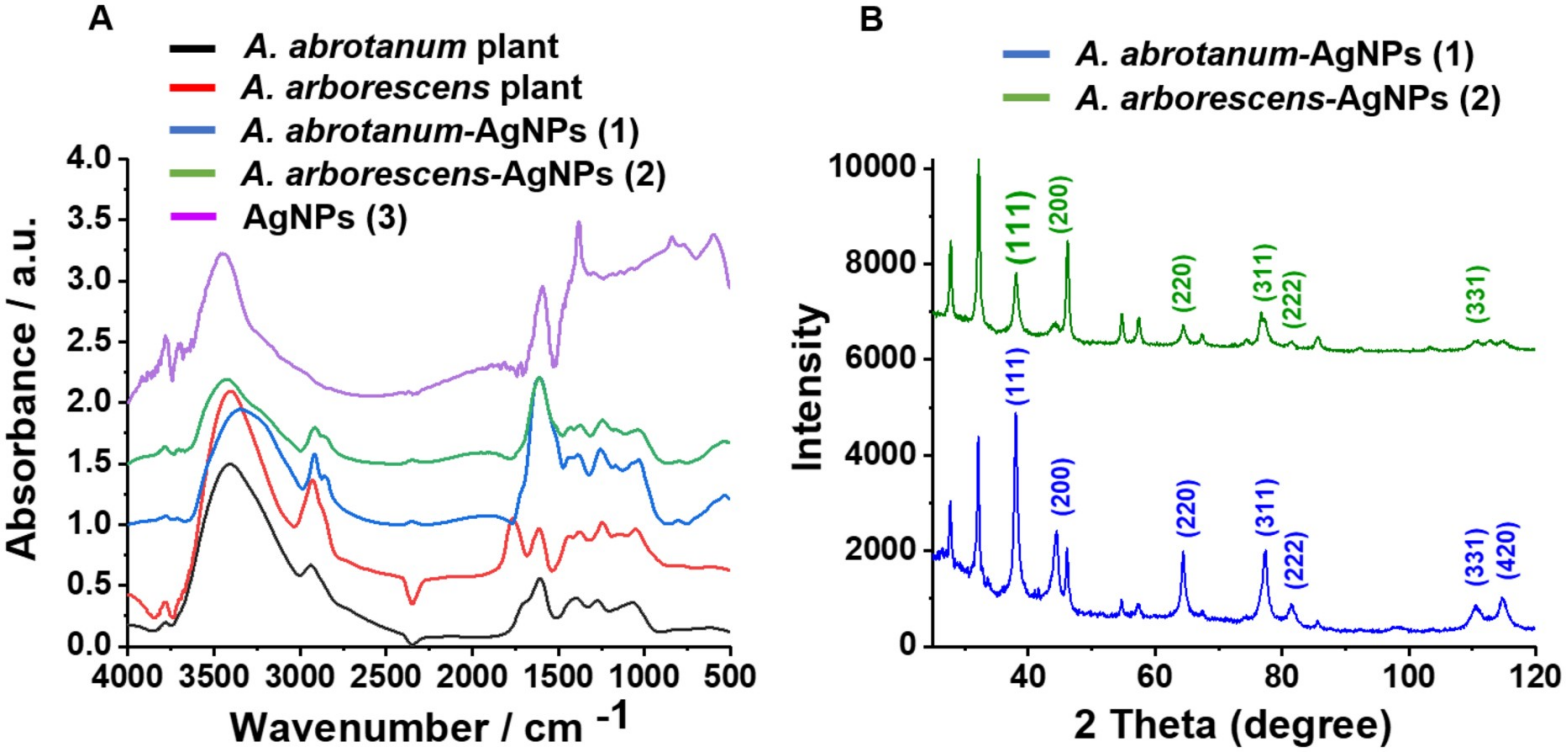
Structural characterization of nanoparticles. A) FT-IR spectra of “green” nanoparticles compared to the spectra of plant extracts and “classical” AgNPs (3). B) XRD pattern of “green” nanoparticles *A*. *abrotanum*-AgNPs (1) and *A*. *arborescens*-AgNPs (2).

FT-IR spectra (Fig 4A) were recorded to evaluate the surface capping of the “green” compared to “classical” nanoparticles. There is a sharp difference in the aspect of the spectra for “green” respect to “classical” AgNPs (3) (Fig 4A), the former showing a number of bands which are not present in the latter, thus confirming the formation of the capping. Such peaks can be tentatively attributed to amines, proteins or polyphenolic compounds of *Artemisia* leaf extracts. The very broad band going from 3100 to 3600 cm^-1^ is encompassing the OH and NH stretching frequencies, the signals around 2900 cm^-1^ are due to the CH stretching mode of hydrocarbon moieties, while those around 1620 (ν C = O) are most probably due to the carbonyl group of amidic compounds (i.e. proteins and enzymes). A series of bands can be attributed to polyphenols: 2920 cm^-1^ (stretching of the C-H bond adjacent to a quinone moiety), 1440 cm^-1^ (stretching of the C = C bond adjacent to the quinone system) and 1250 cm^-1^ (stretching of the C = C bonds of the various coupled aromatic systems). Moreover, the band in the range 1400–1380 cm^-1^ may be attributed to silver nanoparticles in accordance with the literature [44, 49]. As for the AgNPs (3) from classical reduction, the broad band around 3400–3300 cm^-1^ is due to water molecules, while the peaks in the regions 1600–1500 and 1400–1300 cm^-1^ can be attributed to the overlapping of the COO^-^ asymetrical and symmetrical stretching vibrations, respectively, of citrate and ascorbate anions present on the nanoparticle surface [50].

A comparison between the FTIR of the dry plant extracts and the relative AgNPs has been undertaken to add more information about the capping. The two sets of spectra are similar but not identical, showing that most but not all the biomolecules present in the plant extract have contributed to the formation of the capping (Fig 4A).

**Fig 5:**
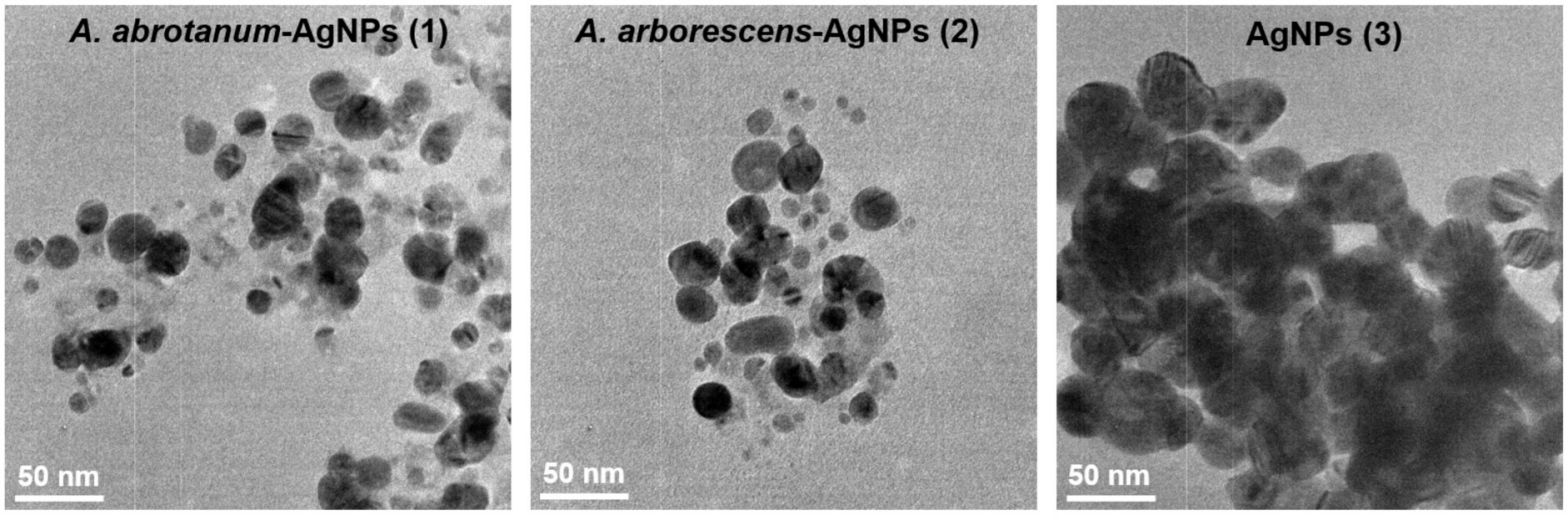
Nanoparticles characterization in size. TEM images of nanoparticles. *A*. *abrotanum*-AgNPs (1) and *A*. *arborescens*-AgNPs (2) have displayed a size range between 20 and 30 nm. Contrariwise, AgNPs (3) have displayed a size greater than green nanoparticles because of the high aggregation observed. TEM micrographs of nanoparticles observed at 50 nm scale.

The XRD patterns showed a progression of broadened line profiles which was matched with the presence of Ag metal phase with space group Fm-3m and a = 4.0853 (4 Ag atoms in the unit cell). In Fig 4B, the crystalline peaks were reported and the XRD data were in agreement with the standard JCPD file no. 04–0783. It should be noted that the pattern reported by Kathoon *et al*. [44] is similar to our recorded progression. The peak profile phase was broadened on account of sensible effects due to crystallite size smallness and lattice strain. These are determined by the Rietveld program to be D = 200 A and e = 0.0023 for Ag. Finally, the samples were prepared for TEM and EDX characterization. Fig 5 shows TEM images of AgNPs, which were close to spherical shape and not fully homogenous in the size range, which anyway appeared to be regular and well below 50 nm in diameter (range 20–30 nm). Moreover, EDX profile confirmed the elemental composition by evidencing the silver peak of each kind of nanoparticle (S1 Fig). The Cu peak also present in the spectrum is due to the grid.

### Hemocompatibility properties of green nanoparticles

**Fig 6:**
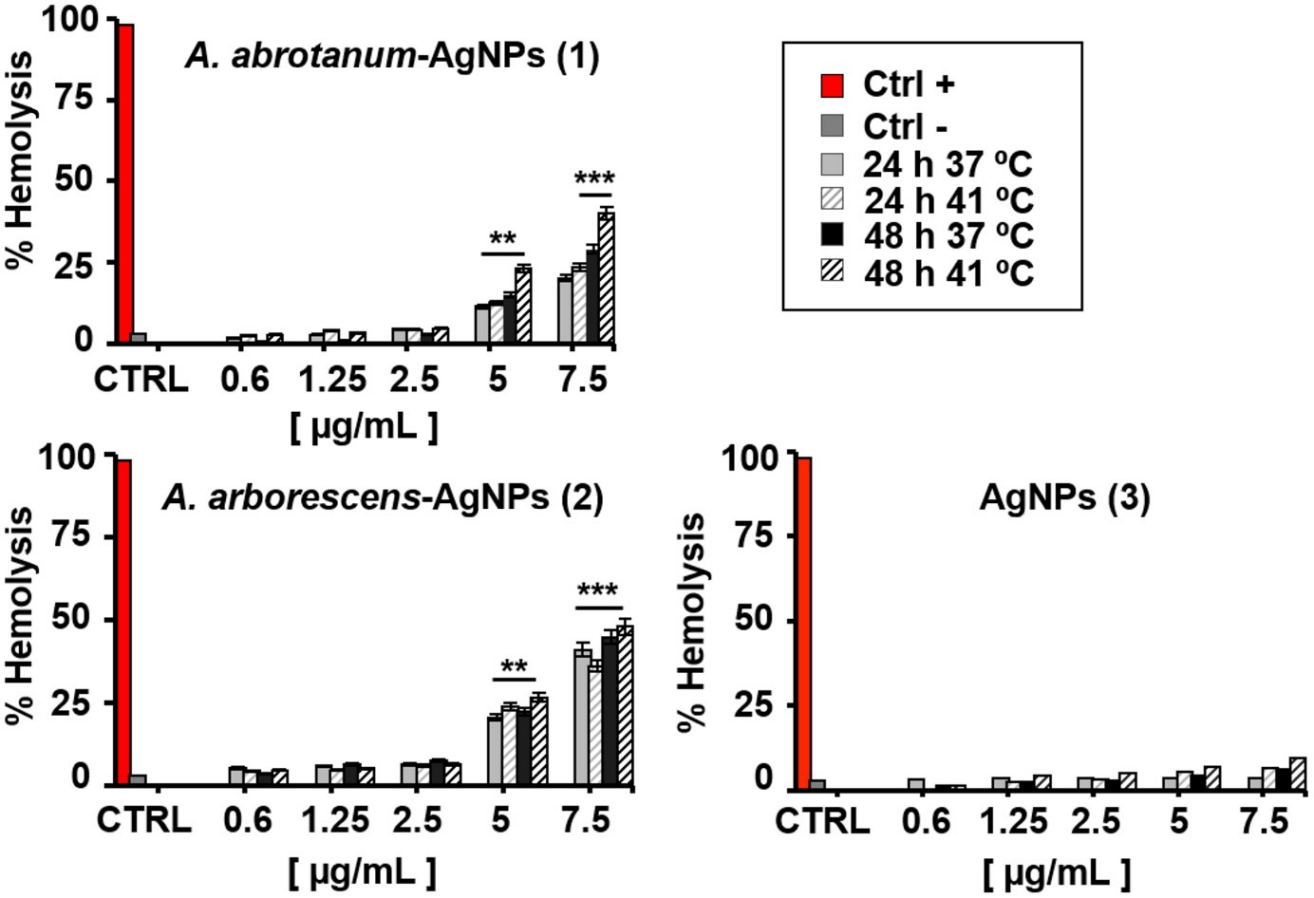
Hemo-biocompatibility assays on human pRBCs. Hemolysis assay on human pRBCs with increasing doses (0.6 μg/mL to 7.5 μg/mL) of “green” nanoparticles and silver nanoparticles treated for 24 and 48 h at 37 and 41 °C. Samples were analysed by spectrophotometer. The percentage of hemolysis is reported in Absorbance (577–655 nm). PBS 1X and 5 mM glucose was used as a negative control (Ctrl-) and hemolysis buffer (5 mmol/L sodium phosphate, 1 mmol/L EDTA, pH 8.0) was used as the positive control (Ctrl+). Statistical significance liken to untreated samples was calculated by Student’s t-test (**, *p* < 0.01), (***, p < 0.001).

The hemolysis assay has commonly been performed at body and fever temperature (37°C and 41°C, respectively) at concentrations ranging from 0.6 to 7.5 μg/mL up to 48 hours of incubation (Fig 6).

Testing both temperature conditions is of particular interest for nanomaterials, as they should be used for therapeutic purposes, which might encounter complex states such as fever [51]. All the tested nanomaterials presented dose-dependent hemolysis on pRBCs after the first 24 hours of incubation, except for AgNPs (3), (Fig 6). At concentration below 5 μg/mL, the hemolytic effect of all tested AgNPs was not significant, as shown in Fig 6. Contrarily, a significant hemolytic activity of the “green” nanoparticles is reported at concentration above 5 μg/mL (*p* < 0.01). In particular, *A*. *arborescens*-AgNPs (2) have shown an evident effect on pRBCs at 5 and 7.5 μg/mL dosage (*p* < 0.01 and 7 μg/mL *p* < 0.001 respectively for both temperature conditions) compared to *A*. *abrotanum*-AgNPs (1), probably due to the different capping and size (Fig 6). Instead, AgNPs (3) did not reveal any significant hemolytic activity in dosages below 7.5 μg/mL at physiological and fever temperature (Fig 6). Otherwise, when measured at fever temperature (41°C) a two-fold increase in hemolysis at 5 and 7.5 μg/mL dosages, compared to lower dosages (0.6, 1.25 and 2.5 μg/mL) of “green” nanoparticles, was observed (Fig 6). In this study, green nanoparticles have shown the hemolytic effects on pRBCs at high dosages (5 and 7.5 μg/mL) due to their small size and their different nature of synthesis. Previous studies have confirmed our findings that the size of AgNPs used in high dosages is a critical factor of hemolysis and especially small sized AgNPs [47], display a great ability to induce hemolysis in pRBCs. Contrariwise, the AgNPs (3) derived from the classical synthesis have shown low activity against parasites and low hemolytic effects probably due to their strong aggregation and their large size. Considering the non-hemolytic activity reported at low dosage of nanoparticles, the next experiments of percentage of parasitemia and morphological study on pRBCs have been performed using the intermediate dosage (2.5 μg/mL).

### Antimalarial activity of “green” nanoparticles on *P*. *falciparum* pRBCs

**Fig 7:**
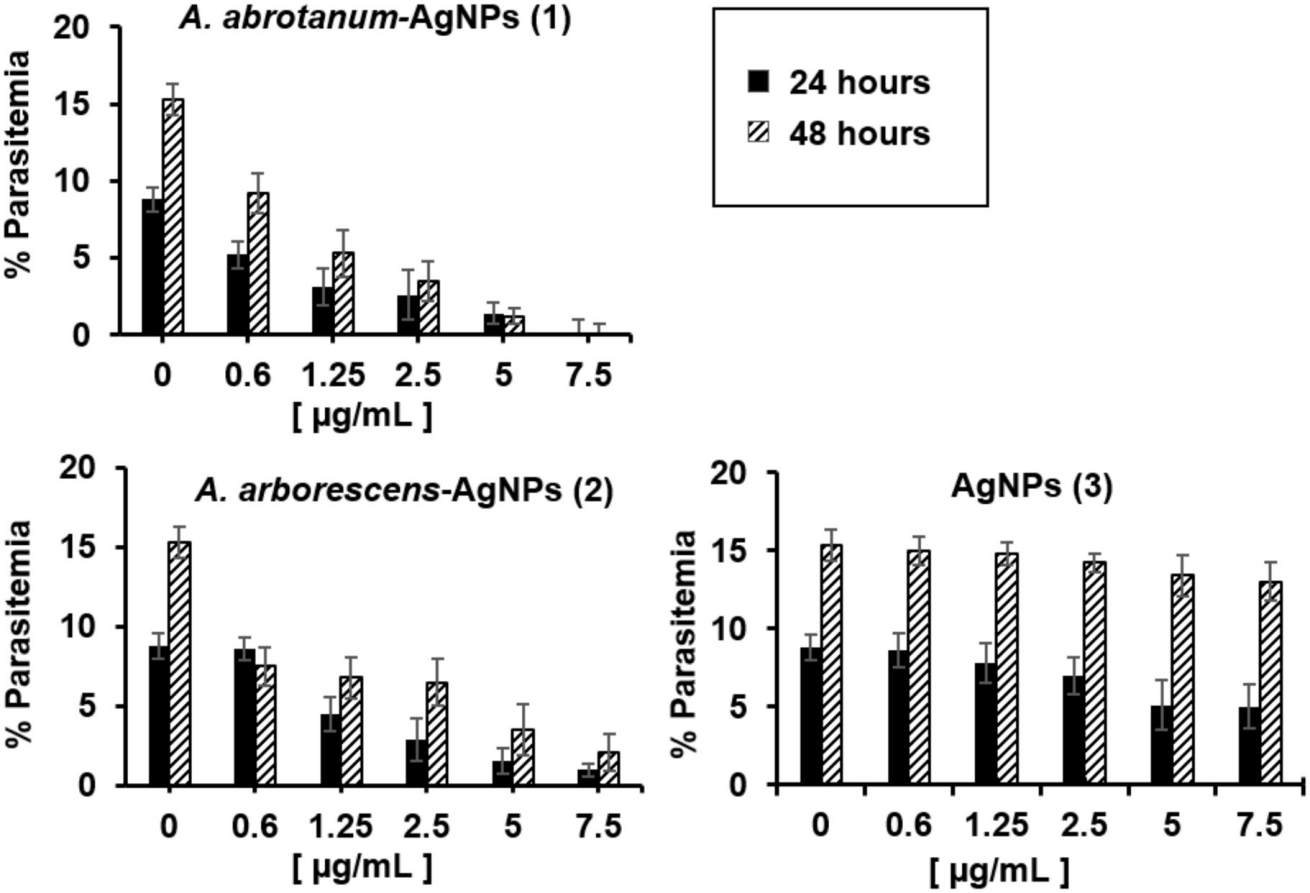
Antiplasmodium effect of nanoparticles. Percentage of parasitemia on pRBCs treated for 24 and 48 h with increasing doses (0.6 μg/mL to 7.5 μg/mL) of nanoparticles.

**Fig 8:**
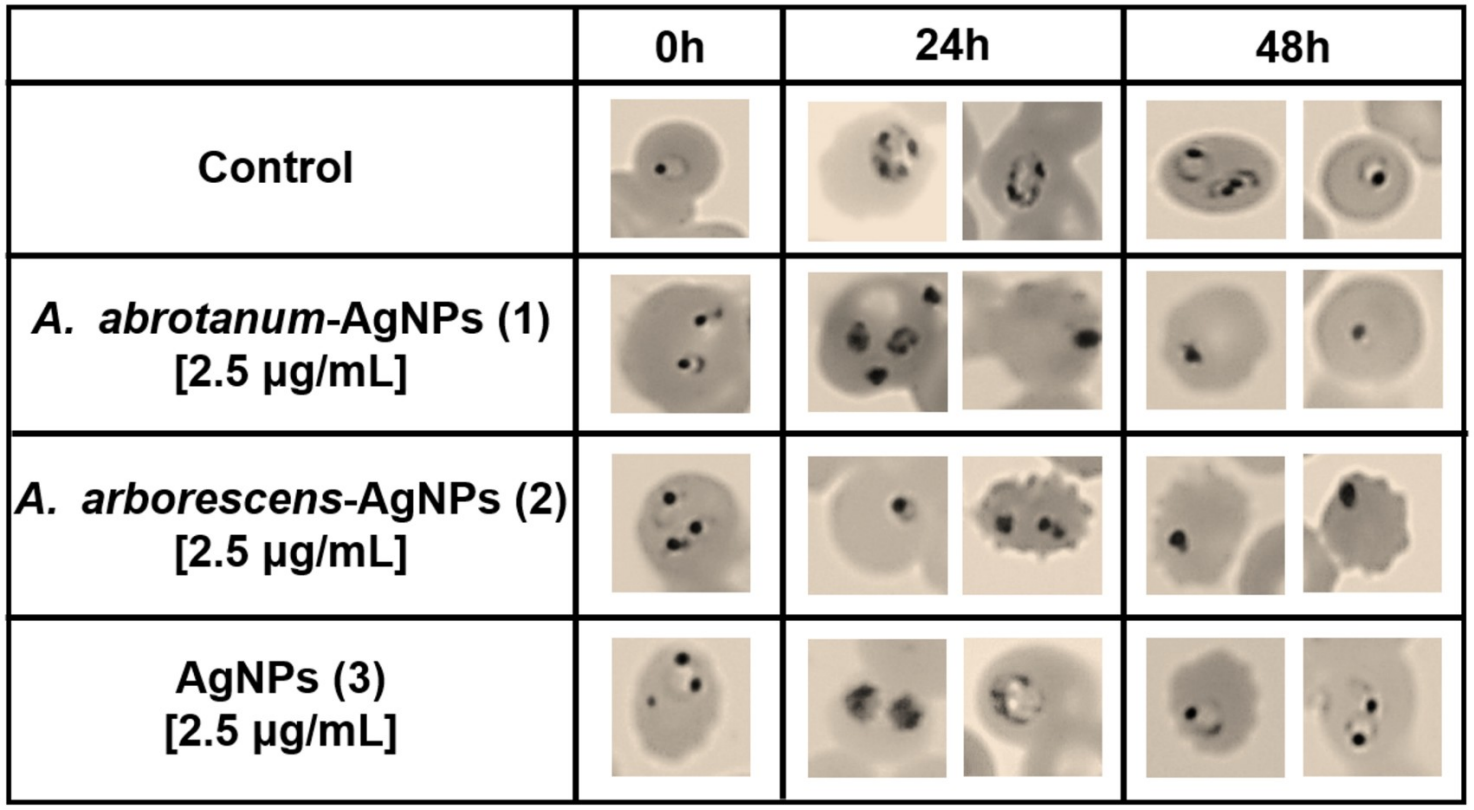
Nanoparticles effect on parasite maturation. The morphology of pRBCs and parasite stage was determined in *P*. *falciparum* strain treated with the intermediate dose of nanoparticles (2.5 μg/mL) after 24 and 48 h of treatment.

The next experiments have been carried out using the non hemolytic concentration of nanoparticles as previously described. In particular, pRBCs were treated with increasing doses of all nanoparticles (0.6 to 7.5 μg/mL) at both 24 and 48 hours in order to evaluate the effect of parasite growth inhibition. Palo Alto (PA) has been used as a representative strain as no significant differences have been observed among the other strains tested (FCB1, It-G and ARS1). *A*. *abrotanum*-AgNPs (1) and *A*. *arborescens*-AgNPs (2) have demonstrated significant *in vitro* activity against *P*. *falciparum* in pRBCs, showing dose-dependent hemolytic effect (Fig 6) connected to the parasite death and a consequent decrease of parasitemia (Fig 7) compared to “classical” AgNPs (3). In particular, these nanoparticles, even at low concentrations (0.6 μg/mL), presented a modest decrease in parasitemia during the first cycle of growth (24 h) and a substantial decrease in parasitemia during the second cycle (48 h) (Fig 7). Considering the evident hemolytic activity of nanoparticles at high concentration, to further evaluate the effects on parasite maturation and death a morphology investigation was performed on PA strain using the most efficient low dosage (2.5 μg/mL). In details, it was found that *A*. *arborescens*-AgNPs (2) showed antiplasmodial activity by blocking the parasite maturation stage from trophozoite to rings (Fig 8). On the contrary, *A*. *abrotanum*-AgNPs (1) have demonstrated their antiplasmodial activity achieving the parasite death compared to the control after 24 and 48 hours of treatment (Fig 8). Moreover, *A*. *abrotanum*-AgNPs (1) showed the best antiplasmodial activity considering the IC_50,90,99_ as reported in Table 1.

**Table 1:** Antimalarial activity of nanoparticles in *in vitro* PA strain.

	*A*. *abrotanum*-AgNPs (1)	*A*. *arborescens*- AgNPs (2)	AgNPs (3)
**IC**_**50**_ **(μg/mL)**	**0.05**	**1.17**	**10.28**
**IC**_**90**_ **(μg/mL)**	**0.08**	**1.45**	**12.81**
**IC**_**99**_ **(μg/mL)**	**0.01**	**1.85**	**16.28**

IC_50/90/99_ concentrations for antimalarial activity in the presence of different nanoparticles.

## Discussion

It is known that a critical step in biomedical applications of nanoparticles is to study their physical-chemical properties in order to correlate their biological activity to specific parameters such as composition, size, shape and capping. In this study, considering the same concentration of nanoparticles used for the analysis, the difference observed during the evaluation of each nanoparticle activity may depend on these parameters. In this context, *A*. *abrotanum*-AgNPs (1) have demonstrated a lower aggregation in aqueous medium and better shape and dimension compared to the other nanoparticles taken into consideration. In order to investigate each AgNPs type effects on PA cultures, their chemical structure has been studied and correlated with their activity and biocompatibility. Indeed, to study the effect of “green” AgNPs on RBCs biocompatibility, several *in vitro* experiments have been performed on human pRBCs infected by *P*. *falciparum*. After the nanoparticles characterization, their hemolytic properties in contact with the human erythrocytes were studied. The increase in hemolytic activity observed is probably due to the higher temperature used for fever simulation, where the RBCs could be sensitive to the temperature of 41°C. On the other hand, based on the large hemolysis dependence on fever temperature, non-hemolytic concentrations were assessed below 5 μg/mL. It is noteworthy that after 24 hours of incubation there was no further increase of the hemolytic activity. The different hemolysis effect could be assigned to the difference in the size of nanoparticles and/or the different biomolecules present in the capping, which cannot be excluded. In fact, it has been reported that the variety of shape, size and chemistry of nanoparticles produce different effects on the biological environment [52].

However, to further evaluate the effects on parasite maturation and death, a morphology investigation was performed on different strains of *P*. *falciparum* pRBCs. The striking morphological alteration observed in fixed blood smears of PA strain treated with *A*. *abrotanum*-AgNPs (1) and *A*. *arborescens*-AgNPs (2), is again most probably linked to the small size and the different nature of synthesis using *Artemisia* extracts. Despite the smaller size of *A*. *arborescens*-AgNPs (2) respect to the other nanoparticles, which should confer them higher efficacy, their lower activity against the parasite may depend on the different capping from the *A*. *arborescens* plant extract used.

Anyway, this method of synthesis has indeed allowed to create small size nanoparticles useful for biomedical applications connected to an effective action against *P*. *falciparum* parasite. In general, all the results gathered in this study underline the higher antiplasmodial efficacy of AgNPs from *A*. *abrotanum* extract respect to the *A*. *arborescens* ones.

## Conclusions

The present work was performed as a pilot study in order to evaluate *Artemisia* sp. derived silver nanoparticles potential antimalarial efficacy in parasitized human red blood cell and to understand their efficacy against *P*. *falciparum* as a new nanotool against malaria. The obtained results have demonstrated that plant extracts derived from *A*. *abrotanum* and *A*. *arborescens* can be used for the facile biosynthesis of silver nanoparticles and to create a bio-capping on the AgNPs useful to modulate their activity against *P*. *falciparum* cultures in *in vitro* experiments. Results evidenced a high anti-malarial activity for *A*. *abrotanum*-AgNPs (1) in compared to *A*. *arborescens*-AgNPs (2). Their hemolytic effect is dose-dependent, and is more pronounced for the smallest AgNPs, i.e. those derived from *A*. *arborescens*, as expected. The high anti-malarial activity for *A*. *abrotanum*-AgNPs (1) is connected to the small size of nanoparticles and their different effect on the parasite cycle. In fact, results underlined that *A*. *abrotanum*-AgNPs (1) were been able to hinder the stage of the maturation of the parasite locking it in the ring stage after treatment. The mechanism behind this behaviour is still unknown. Studies reporting the biological bases of antiprotozoal action of silver nanoparticles are very rare [53], so the details on the processes leading to their antiplasmodium effects are lacking and proper investigation should be undertaken as soon as possible in order to cover this important topic and allow a more efficacious research to fight malaria with these new nanoweapons. Anyhow, the study on AgNPs antibacterial properties unveiled some relevant aspects of their activity, which can be mainly connected to the generation of ROS (reactive oxygen species) as the promoter of cell death mechanisms, especially via mitochondrial apoptotic pathways, together with extensive damage to the cell membrane and enzyme deactivation via silver binding (17). All these events, alone or combined, could be able to disrupt the functions of plasmodium cells, leading to the consequences observed in our experiments. Considering the data presented in this study, the antiplasmodial activity of *Artemisia*-derived AgNPs in *in vitro* experiments against *P*. *falciparum* is promising and deserves further research.

## Supporting information

S1 Fig Elemental composition of “green” AgNPs. Evaluation of nanoparticles silver signal using EDX profile of *A*. *abrotanum*-AgNPs (1), *A*. *arborescens*-AgNPs (2) and AgNPs (3). Cu peaks presented in the graphs are due to the grid used.
(TIF)

S2 Fig Zeta potential analysis. Evaluation of *A*. *abrotanum*-AgNPs (1) stability and dispersion in aqueous medium.
(TIF)

S3 Fig Zeta potential analysis. Evaluation of *A*. *arborescens*-AgNPs (2) stability and dispersion in aqueous medium.
(TIF)

S4 Fig Zeta potential analysis. Evaluation of AgNPs (3) stability and dispersion in aqueous medium.
(TIF)
