# Supplementary figures and images for: The potential antimalarial efficacy of hemocompatible silver nanoparticles from Artemisia species against P. falciparum parasite
Source: PLoS One. 2020 Sep 1;15(9):e0238532. doi: 10.1371/journal.pone.0238532 (PMC7462267; doi:10.1371/journal.pone.0238532)

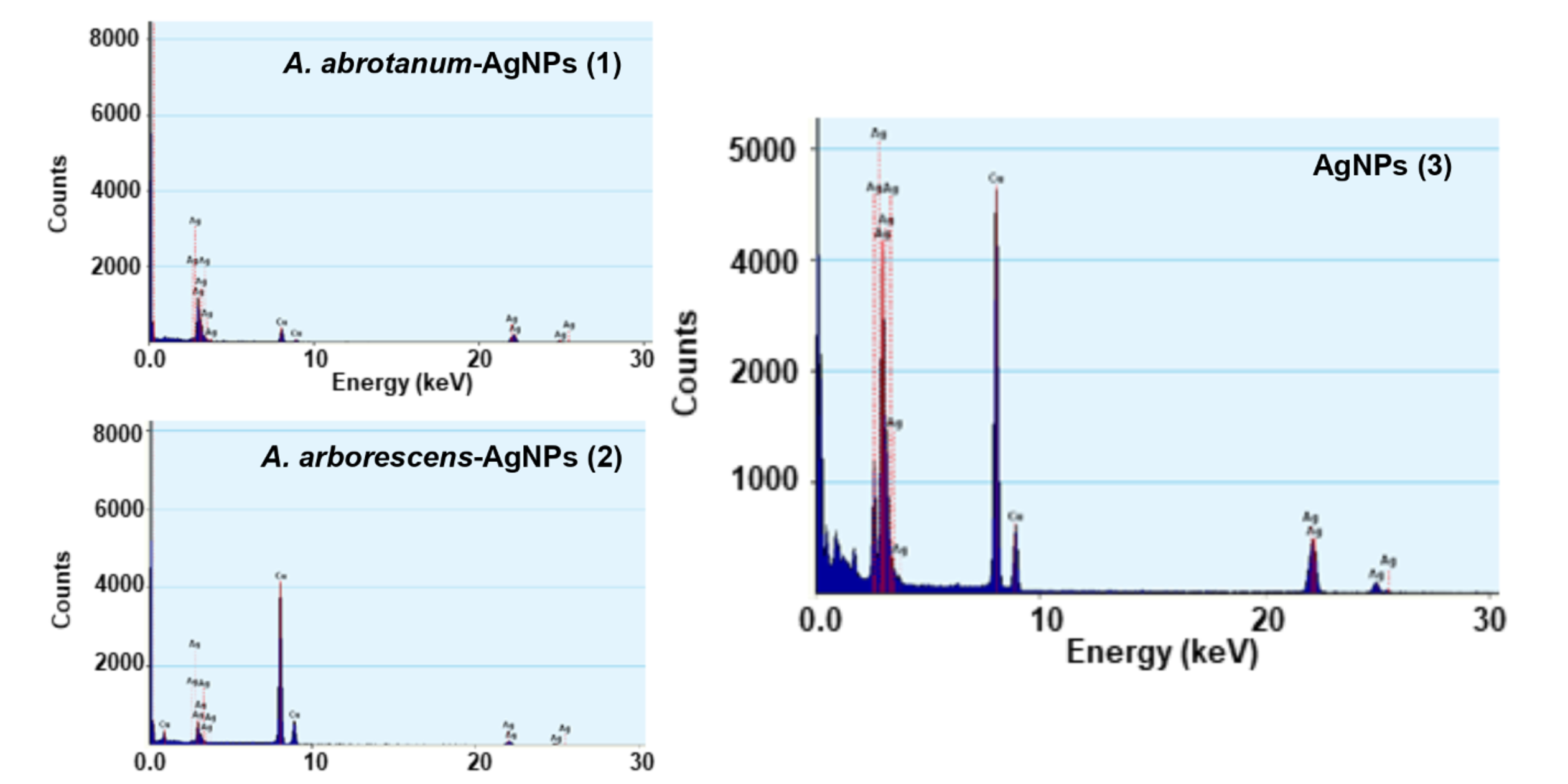

Supplement: S1 Fig — Evaluation of nanoparticles silver signal using EDX profile of A. abrotanum-AgNPs (1), A. arborescens-AgNPs (2) and AgNPs (3). Cu peaks presented in the graphs are due to the grid used. (TIF) [file pone.0238532.s001.tif]

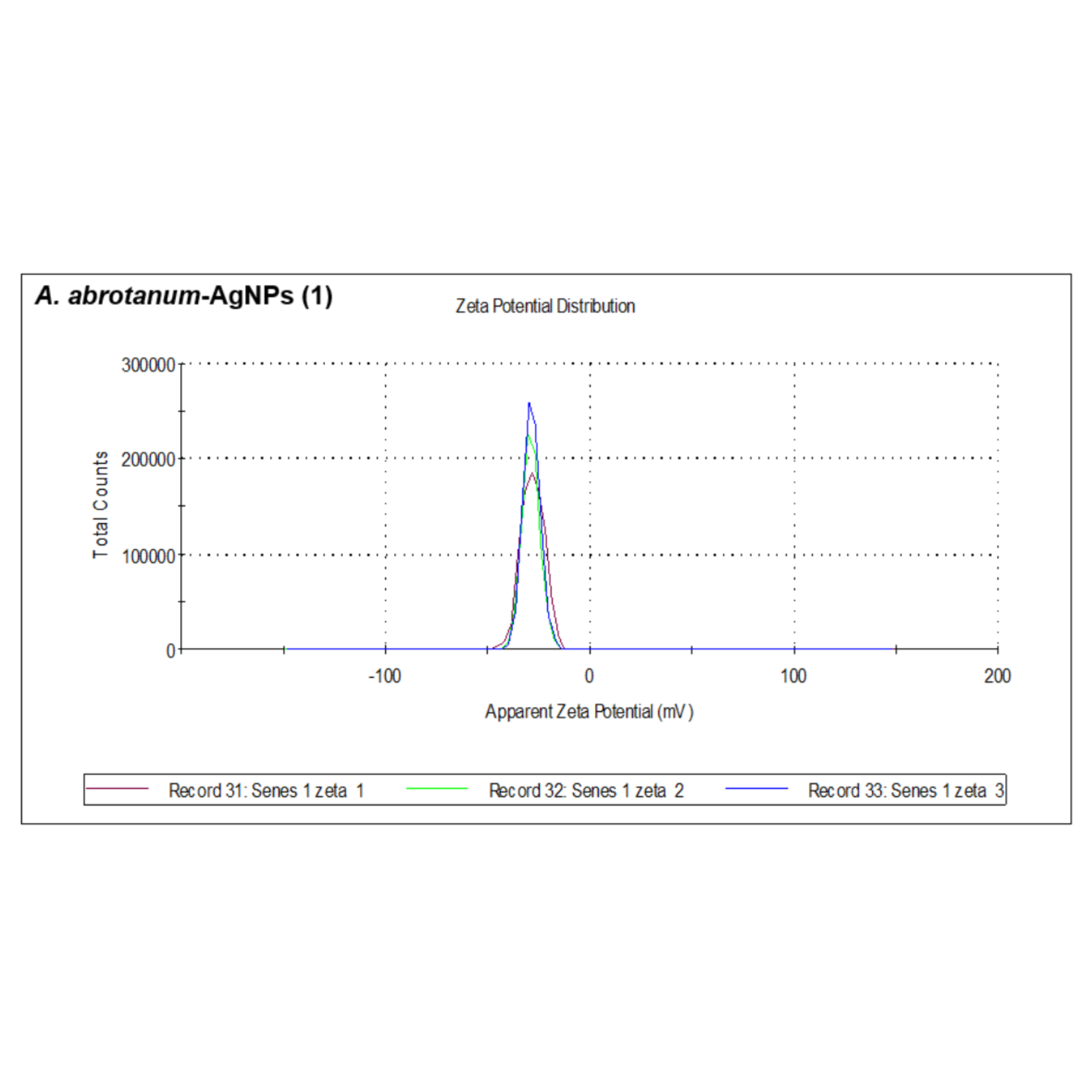

Supplement: S2 Fig — Evaluation of A. abrotanum-AgNPs (1) stability and dispersion in aqueous medium. (TIF) [file pone.0238532.s002.tif]

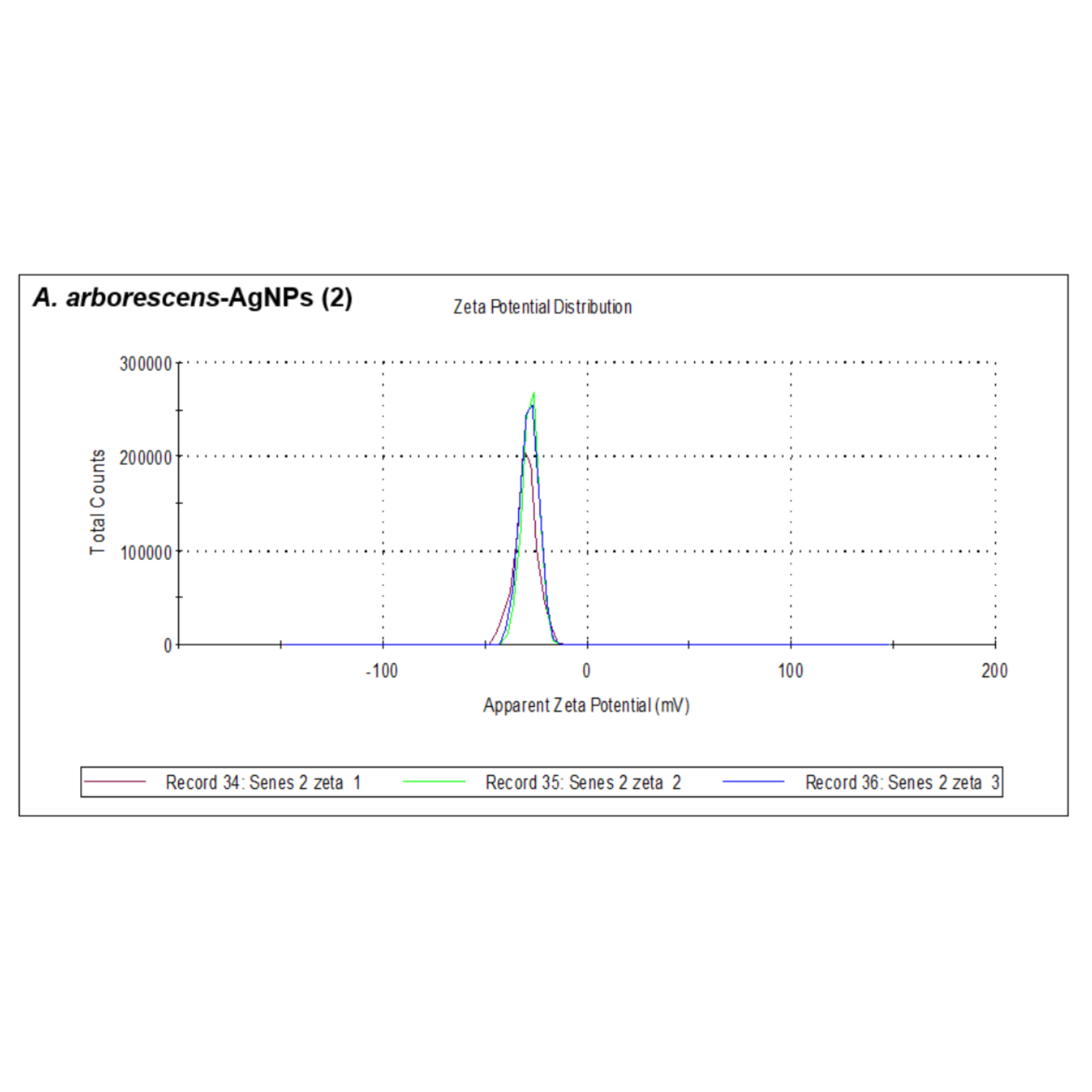

Supplement: S3 Fig — Evaluation of A. arborescens-AgNPs (2) stability and dispersion in aqueous medium. (TIF) [file pone.0238532.s003.tif]

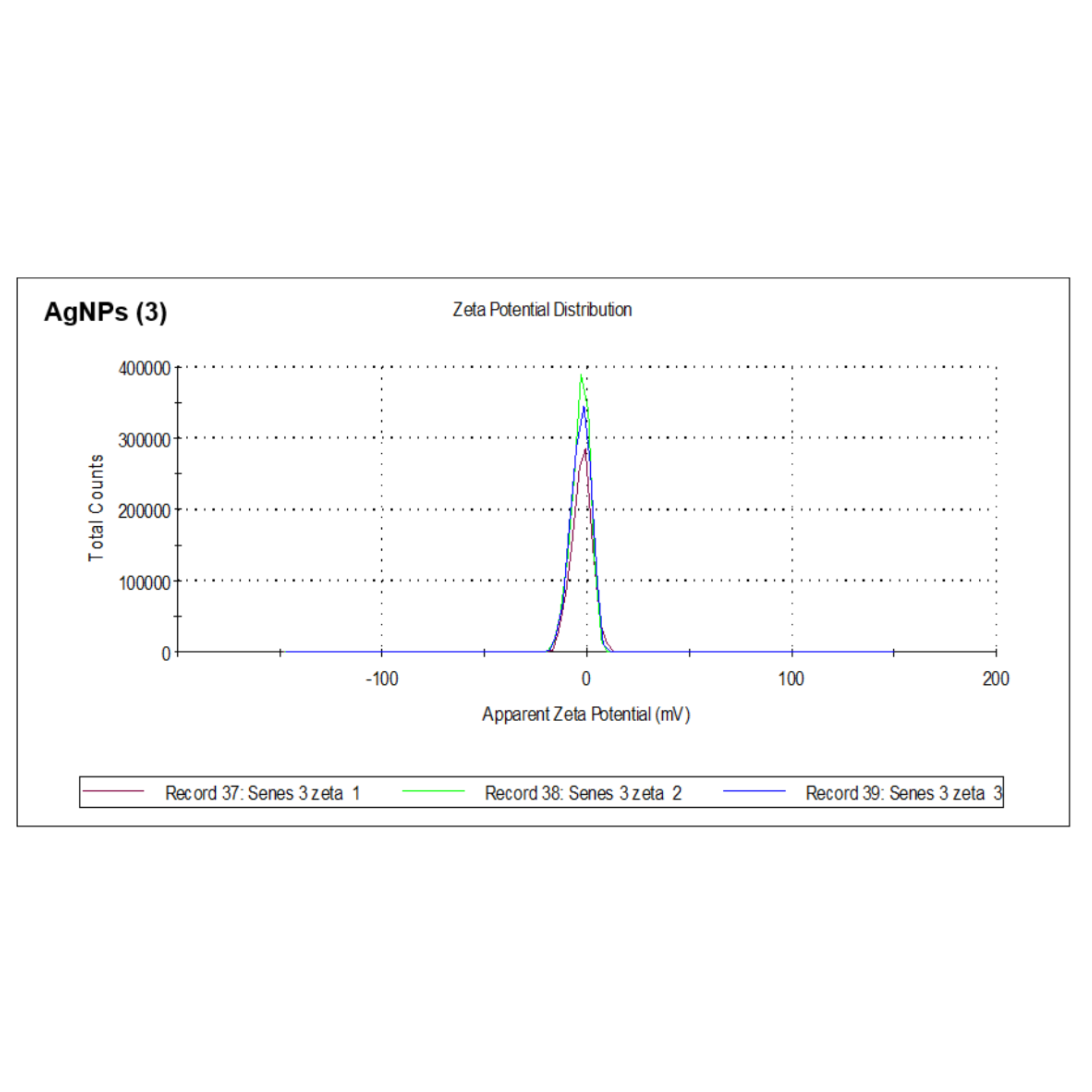

Supplement: S4 Fig — Evaluation of AgNPs (3) stability and dispersion in aqueous medium. (TIF) [file pone.0238532.s004.tif]
